# Examining the complexity of the relationship between deviant peers affiliation and child-to-parent violence in young adults: the mediating role of drug use and the moderating role of family support

**DOI:** 10.3389/fpsyg.2026.1814315

**Published:** 2026-06-04

**Authors:** Lourdes Contreras, María J. Navas-Martínez, M. Carmen Cano-Lozano

**Affiliations:** Department of Psychology, University of Jaén, Jaén, Spain

**Keywords:** child-to-parent violence, deviant peers, drug use, family support, young adults

## Abstract

**Introduction:**

Research on child-to-parent violence has expanded considerably over the last decade. Most of the studies have been mainly conducted with samples of adolescents and very few has explored this issue in young adults. However, this form of violence is not limited to adolescence but continues into young adulthood. Despite the extensive literature on family and individual risk factors, some variables remain underexplored, especially social variables such as deviant peer affiliation. This study is aimed to explore, in a sample of non-emancipated young adults, the relationship between deviant peer affiliation and child-to-parent violence. On the one hand, it is examined the mediating role of drug use in this relationship and, on the other hand, the moderator role of family support.

**Methods:**

The sample consisted of 1,147 young adults (48% women) aged between 18 and 25 years. The instruments included the Child-to-Parent Violence Questionnaire, and *ad hoc* Deviant Peers Scale, the Scale of Tobacco, Alcohol and Other Drug Use and the Multidimensional Scale of Perceived Social Support.

**Results and discussion:**

The results indicated that deviant peer affiliation is positively and indirectly related to child-to-parent violence through drug use. Furthermore, family support moderated the positive relationship between deviant peer affiliation and CPV, reducing its magnitude. These findings provide additional evidence of the mechanisms mediating the relationship between deviant peer affiliation and child-to-parent violence, with drug use acting as a mediator. The results also highlight the protective role of family support against deviant peer influence, with implications for prevention and intervention programs for this type of violence.

## 1 Introduction

Child-to-parent violence (CPV hereinafter) has been conceptualized as any act of a child that is intended to cause physical, psychological or financial damage to gain power and control over parents (Cottrell, 2001) and dominate them (Howard and Rottem, 2008) in a conscious and intentional manner, and excluding isolated episodes of violence (Pereira et al., 2017). Previous research has also highlighted the heterogeneity of CPV, including reactive, impulsive, or defensive forms of violence (e.g., Ibabe, 2020; Simmons et al., 2018). The incidence rates of this type of violence have dramatically increased in the last decade across multiple countries around the world. In this regard, most of studies have been conducted with adolescent samples and very few with young adults (Cano-Lozano et al., 2021b). Unfortunately, CPV does not disappear when children reach adulthood, and the increased autonomy, power of intimidation and physical strength may amplify the potential for harm in young adulthood, compared to earlier stages. In fact, the legal consequences for adult perpetrators are more severe than for minors (Ibabe, 2020). So far, there is little research that includes offenders over the age of 18, who are legally considered adults. Data from different countries reveal that at least half of young adults between 18 and 25 continue living with their parents, and warn that this may be a phenomenon of growing importance given the increasingly older age at which young people become independent in Western countries (Simmons et al., 2018).

The scarce studies with young adults reveal a high frequency of this type of violence. For instance, research suggests that as many as 20% of Australians young adults between 18 and 24 years of age have engaged in some violent behavior toward their fathers, with up to 16% exhibiting similar behavior toward their mothers (Simmons et al., 2020). By types of CPV, a study conducted in Sweden with 687 parents of children over 18 years found that 19% of parents admit to experiencing physical CPV and about 40% financial CPV (Johnson et al., 2022). Recent studies from Spain, Italy and Ecuador reported that the percentages of psychological violence towards parents oscillates between 43.9% and 60.3%, physical violence range between 1.9% and 2.5%, financial violence oscillates between 14.1% and 36.7% and finally, control/domain behaviors range between 37% and 68% (Burgos-Benavides et al., 2025; Cano-Lozano et al., 2021b; Navas-Martínez et al., 2025a).

As with other social problems, the dramatic increase in this type of youth violence has led to a notable and consequent increase in research on this topic and the proliferation of theoretical frameworks, in order to understand the underlying factors of CPV. In this regard, the ecological models are the most solid approaches to explain this phenomenon (Cottrell and Monk, 2004; Hong et al., 2012). These models propose that there are multiple levels of influence concerning CPV, highlighting the reciprocal interactions of these levels. Thus, there are diverse variables, from the immediate setting of the individual to a broader cultural and social context.

As previously mentioned, there are very few studies on risk factors for CPV with young adults, but the existent research with adolescents reveals that, at individual level, some of them stand out, such as emotional and socio-cognitive difficulties (Beckmann et al., 2017; Cano-Lozano et al., 2020; Contreras et al., 2020, 2025; Martínez-Ferrer et al., 2018; Orue et al., 2019) and attitudes that justify the use of violence, among others (Cano-Lozano et al., 2023; Contreras et al., 2020, 2025; Orue et al., 2019). Furthermore, some studies have shown that drugs use is positively related to CPV (Beckmann et al., 2017; Cano-Lozano et al., 2020) not only during adolescence but also during adulthood (Svensson et al., 2019). In general, the relationship between drug use and violence is supported by empirical evidence (Lim and Lui, 2016; Sontate et al., 2021). Notwithstanding, the strength and nature of this association vary substantially depending of other variables such as the type of substance, individual characteristics and contextual factors (Ogden et al., 2025; Rafiei and Kolla, 2022; Stoddard et al., 2020), suggesting a complex and multifactorial rather than purely causal relationship. In the context of CPV, Simmons et al. (2018) noted that in community samples the effect sizes were small and, in forensic samples, the drug use rates were similar to those of offenders in general. This could indicate that, although drug use clearly contributes to the occurrence of conflicts between parents and children, drugs use is part of an underlying pattern of antisocial behavior rather than a specific causal factor in CPV (Armstrong et al., 2018; Contreras and Cano-Lozano, 2015; Simmons et al., 2018). For this reason, further research is need in order to clarify the role of drug use in CPV cases.

Variables related to the social context have been scarcely analyzed in the field of CPV, being particularly noteworthy the violence exposure in the school or in the street (Contreras and Cano-Lozano, 2016). Another relevant social variable is the affiliation with deviant peers, which is a consistent risk factor for the antisocial behavior during adolescence (Gallupe et al., 2019; Sijtsema and Lindenberg, 2018). In the case of CPV, there are not much research about its role in this type of violence, although some studies have found that adolescents who assault their parents tend to relate with deviant peer groups (Contreras and Cano-Lozano, 2015; Loinaz and de Ma Sousa, 2020). The peer group, as a context for socialization, may serve as a behavioral model in which violence is used to control and dominate others. Consequently, adolescents who learn these violent behaviors often apply them in their interactions with their parents (Cottrell and Monk, 2004). Furthermore, according to classical interactional theory of delinquency (Thornberry, 1987; Thornberry et al., 1994), these associations are bidirectional and dynamic (Cho, 2021; Kim and Lee, 2023), as affiliation with deviant peers may promote violent behaviors toward parents, while CPV itself fosters the consolidation of bonds with such peers, creating a reciprocal process of influence and maintenance.

However, some recent studies have deepened into this issue indicating that the deviant peer affiliation does not directly trigger CPV, but through other variables. In this line, according to Véronneau and Dishion (2010), a key yet often overlooked aspect of the bioecological model (Bronfenbrenner and Morris, 2006) is the idea that interrelationships between variables may play a more critical role in understanding human development than direct relationships. From this premise, the association between different significant variables from various socialization contexts could better explain some behaviors than those variables separately. Concretely, in studies with adolescent samples, it has been found an indirect effect of deviant peer affiliation on CPV through drug use (Cano-Lozano et al., 2020; Del Hoyo-Bilbao et al., 2020). For this reason, it could be interesting to deepen into the relationship between deviant peer affiliation and CPV by examining the mediator role of drug use.

In addition, in previous studies the affiliation with deviant peers was influenced by family variables such as a lack of parental support, parental inefficiency (Del Hoyo-Bilbao et al., 2020) or low perceived parental warmth (Cano-Lozano et al., 2020). In this regard, the family support is defined as the set of emotional, instrumental, and relational resources perceived as available within the family system as a whole, including siblings and other family members, and not exclusively parental figures (Caplan, 1974; Cohen and Wills, 1985; Thoits, 2011). This support is expressed through affection, understanding, communication, and a sense of belonging, and constitutes a key protective factor against affiliation with deviant peers, antisocial behavior, and violence within the family context (Cottrell and Monk, 2004; Dishion and Tipsord, 2011; Holt, 2016). In previous studies the family support has been shown to moderate between different risk factors and several problems during adolescence (Gao et al., 2020; Kennedy et al., 2010; Schofield et al., 2012; Ozer, 2005) buffering, for example, the influence of deviant peer affiliation on conduct problems and delinquency (Gao et al., 2013; Trudeau et al., 2012). Overall, these findings suggest that adolescents embedded in cohesive and supportive family context may be less susceptible to the influence of antisocial peer norms. Therefore, it could be also valuable to further explore the association between deviant peer affiliation and CPV by analyzing whether family support acts as a moderating factor in this relationship.

In view of the previous literature and particularly, given the scarcity of studies on CPV conducted with non-emancipated (living with their parents) young people (over 18 years old), the purpose of the current study was to examine the mediating and the moderating variables that intervene in the relationship between deviant peer affiliation and the CPV in a sample of young adults. Concretely, the first objective was to analyze whether drug use mediates the relationship between deviant peer affiliation and the violence towards fathers and mothers. The second objective was to examine whether family support moderates this relationship (between deviant peer affiliation and violence towards fathers and mothers).

The hypotheses were as follows:

*H1*: Deviant peer affiliation was expected to be positively and directed related to CPV, and indirectly more strongly through drug use (Cano-Lozano et al., 2020; Del Hoyo-Bilbao et al., 2020).

*H2*: No specific hypotheses are established given the lack of evidence in the field of CPV. Based on results from other fields of study, family support is expected to buffer the positive relationship between deviant peer affiliation and CPV (Gao et al., 2013; Trudeau et al., 2012).

## 2 Materials and methods

### 2.1 Participants

A total of 1,147 non-emancipated (living with their parents) young people (48% women and 52% men) aged between 18 and 25 years (*M_age_* = 21.4, *SD* = 1.9) participated in the study. The inclusion criteria were: (a) being between 18 and 25 years old, and (b) regularly living with at least one parent during the previous year. Participants who were not living with their parents on a regular basis were excluded. Of the total sample, 50.8% were university students and 49.2% were non-university students, most of them (85%) from Andalusia (a region in the south of Spain), with the remainder coming from other Spanish provinces. Regarding the family structure, 79% came from nuclear families, 12.3% from single-parent families (mother only), 2.9% from single-parent families (father only), and 5.8% from reconstituted families.

### 2.2 Instruments

Participants provided information on sociodemographic data (age, sex, academic degree) and family data (marital status of parents).

*Child-to-parent Violence Questionnaire, youth version* (CPV-Q, Cano-Lozano et al., 2021a). It consists of 19 parallel items assessing various forms of CPV -physical, psychological, financial, and control/domain behaviors- directed separately toward the father (*α* = 0.841; α_o_ = 0.808; *ω* = 0.906; ω_o_ = 0.812) and the mother (*α* = 0.843; α_o_ = 0.808; ω = 0.912; ω_o_ = 0.812). Participants reported the frequency of these behaviors over the last year using a 5-point scale ranging from 0 (never occurred) to 4 (occurred six or more times). This instrument also includes a second part to assess the reasons for CPV (instrumental or reactive), although in the present study, CPV was operationalized independently of the specific underlying motivation. The CPV-Q has been used in multiple international studies, showing adequate psychometric properties, as well as good reliability and validity of evidence (e.g., Burgos-Benavides et al., 2025; Navas-Martínez et al., 2025a).

*Ad hoc Deviant Peers Scale* (based on the Peers Deviant Scale of Barnow et al., 2005). The participant has to answer how many of their friends use drugs, exhibit violent behavior, or have been involved in criminal activity in the last year (3 items: *α* = 0.661; α_o_ = 0.808; *ω* = 0.683; ω_o_ = 0.812) using a 4-point scale ranging from 0 (none of them) to 3 (all of them).

*Scale of Tobacco, Alcohol and Other Drug Use* (del García Castillo-López, 2011, in del García Castillo-López, 2011, p.10). We use the part of the scale that assesses the frequency of consumption of a total of nine toxic substances during the last year (9 items, α = 0.605; α_o_ = 0.868; ω = 0.804; ω_o_ = 0.932), with a 5-point scale ranging from 0 (never) to 4 (daily). Previous studies report appropriate psychometric properties of this instrument (e.g., Aragón Luna et al., 2019).

*Multidimensional Scale of Perceived Social Support* (MSPSS, Zimet et al., 1988; Spanish validation, Landeta and Calvete, 2002). This scale evaluates the social support that the participant perceives from their friends, family, and other relevant people during the last year. In this study only the family support dimension is included (4 items: α = 0.932; α_o_ = 0.960; ω = 0.954; ω_o_ = 0.973) with a 6-point scale ranging from 1 (totally disagree) to 6 (totally agree). This instrument has shown excellent psychometric properties in previous studies (e.g., Calderón et al., 2021; López-Angulo et al., 2021).

### 2.3 Procedure

For this research, we requested and obtained a favorable report from the Ethics Committee of the University of Jaén, Spain (Ref. ABR.22/5.PRY). The initial recruitment of the sample was carried out through collaboration with university students who were invited to participate in the study. Subsequently, snowball sampling was used to complete the sample. In this method, initial participants were asked to invite their personal contacts to recruit additional participants (Jackson et al., 2003). Prior to data collection, participants were informed of the study objectives and provided informed consent. Each participant was assigned a unique identification code to ensure data confidentiality. Participation was voluntary, and no incentives were provided for participation in the study. The questionnaires were administered online using the Google Forms platform.

### 2.4 Data analyses

In a preliminary phase, several reliability estimates were calculated (Cronbach’s alpha, ordinal alpha, McDonald’s omega, and ordinal omega); given the ordinal nature of the items, the coefficients based on polychoric correlations (ordinal alpha and ordinal omega) were the most appropriate indicators of internal consistency in this study (Dunn et al., 2014; Flora, 2020). Next, descriptive statistics for the study variables were calculated, and Spearman’s correlation analyses were performed.

Second, simple mediation and moderation tests were conducted using the PROCESS 4.1 macro (Model 4 for mediation and Model 1 for moderation). Regarding the mediation analysis, we examined the direct effect of deviant peer affiliation (predictor variable) on the CPV toward fathers and mothers separately (dependent variables). Subsequently, we analyzed the indirect effect of drug use (mediator variable) in the relationship between deviant peer affiliation and CPV. Regarding the moderation analysis, we evaluated whether the relationship between deviant peer affiliation (predictor variable) and CPV toward fathers and mothers separately (dependent variables) varied depending on levels of family support (moderator variable). All statistical assumptions were satisfied, with the exception of normality, a common issue in research on violent behaviors in community-based samples. Since a non-normal data distribution can increase the probability of Type I and Type II errors, this study employed the nonparametric percentile bootstrap method with bias correction to assess the significance of the regression coefficients (Erceg-Hurn and Mirosevich, 2008). We drew 10,000 bootstrap samples to estimate the standard errors of the parameter coefficients and generate 95% bootstrap confidence intervals (Hayes, 2017). Effects were considered statistically significant when the confidence interval did not include zero (Alfons et al., 2022; Preacher and Hayes, 2008).

Third, despite the robustness of the nonparametric bootstrap method under skewed distributions, robustness analyses were conducted using negative binomial models to examine the potential impact of non-normality on the main results. Although the dependent variable in this study is not a strict count, this technique allows us to observe the performance of the effects under a distribution that accounts for overdispersion and the floor effect in the data. These results were compared with the main analyses to assess the robustness of the findings.

## 3 Results

Table 1 shows the descriptive statistics of the variables, as well as the correlations among them. The results show that all study variables are significantly correlated (see Table 1). CPV toward father is positively associated with deviant peer affiliation (*ρ* = 0.217) and drug use (*ρ* = 0.241), and negatively associated with family support (*ρ* = −0.263). Similarly, CPV toward mother is positively related to deviant peer affiliation (*ρ* = 0.223) and drug use (*ρ* = 0.245), and negatively related to family support (*ρ* = −0.300). Deviant peer affiliation is also positively correlated with drug use (*ρ* = 0.341) and negatively with family support (*ρ* = −0.124), while drug use shows a negative correlation with family support (*ρ* = −0.111).

**Table 1:** Descriptive statistics and Spearman correlations between the study’s variables.

Variables	M ± SD	Min	Max	1	2	3	4	5
1. Child-to-Father Violence	6.109 ± 6.249	0	46	—				
2. Child-to-Mother Violence	6.574 ± 6.327	0	62	0.776	—			
3. Deviant peer affiliation	0.998 ± 1.332	0	9	0.217	0.223	—		
4. Drug Use	4.708 ± 3.744	0	36	0.241	0.245	0.341	—	
5. Family Support	19.657 ± 4.838	4	24	−0.263	−0.300	−0.124	−0.111	—

*N* = 1,147.

All correlations are significant at *p* < 0.001.

**Figure 1:**
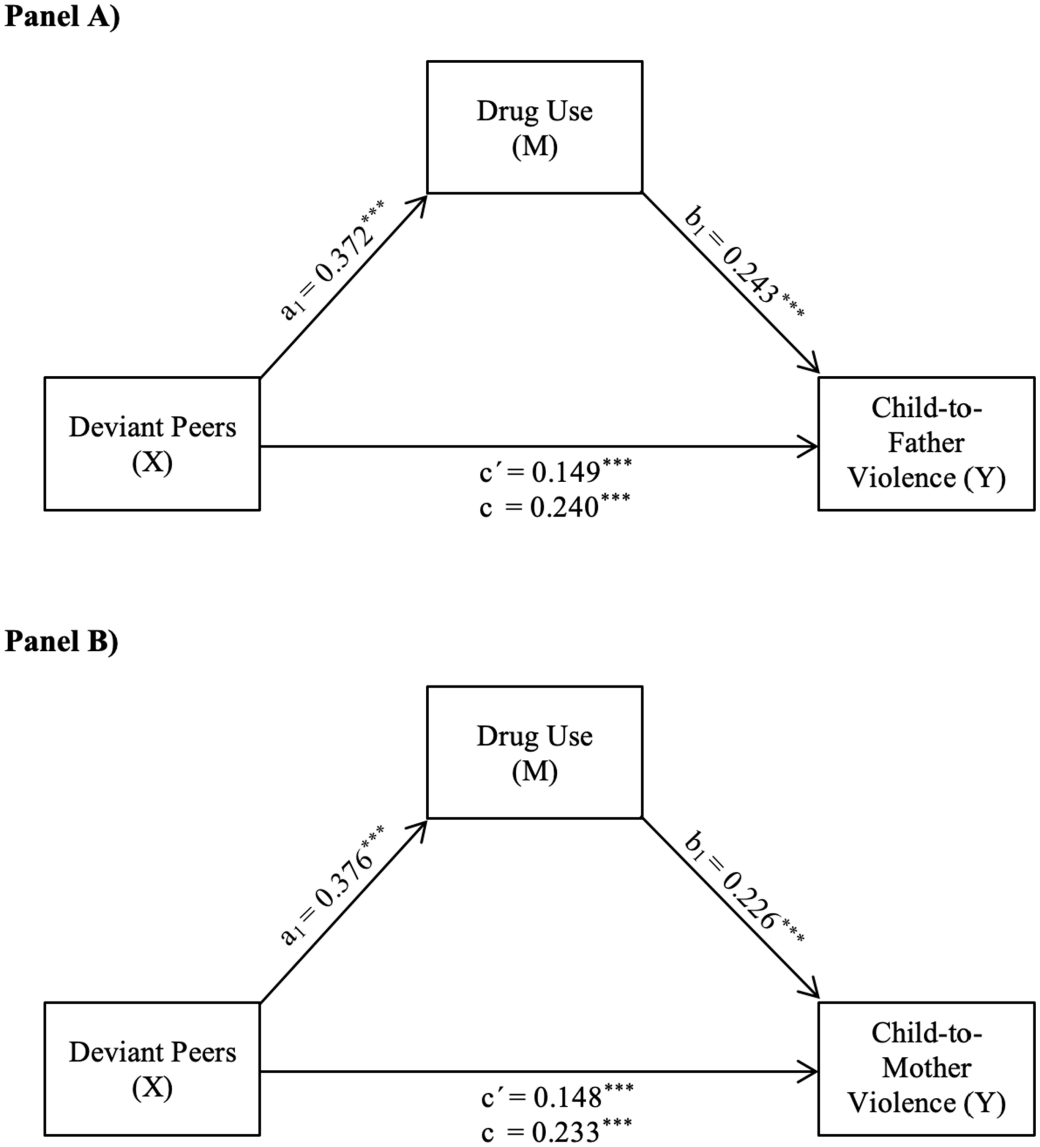
Statistical mediation model. The model for violence towards fathers is presented in panel **(A)**, and the model for violence towards mothers is presented in panel **(B)**; c’ = direct effect; c = total effect. ***p < 0.001.

First, the proposed mediation models were tested. As shown in Figure 1, deviant peer affiliation significantly predicts CPV toward both the father (Panel A) and the mother (Panel B). However, the results further indicate that drug use partially mediates this relationship, with the indirect path through drug use being stronger than the direct path in both models. This indicates that the total effect of the association between the deviant peer affiliation and CPV is larger than the direct effect because a substantial portion of this association is explained indirectly through drug use.

In particular, the child-to-father violence model (see Table 2) shows that deviant peer affiliation is significantly and directly related to CPV (*β* = 0.149, *p* < 0.001, 95% CI [0.415, 0.990]). In addition, a significant indirect effect through drug use was observed (*β* = 0.090, *p* < 0.001, 95% CI [0.242, 0.665]), which accounted for 37.5% of the total effect of deviant peer affiliation on CPV toward the father (*β* = 0.240, *p* < 0.001, 95% CI [0.854, 1.402]). This indirect association operated through a significant pathway in which deviant peer affiliation predicted higher levels of drug use (*β* = 0.372, *p* < 0.001, 95% CI [0.898, 1.214]), which in turn predicted higher levels of CPV toward the father (*β* = 0.243, *p* < 0.001, 95% CI [0.302, 0.505]).

**Table 2:** Coefficients for the mediation models of the relationship between deviant peer affiliation and child-to-parent violence.

Effect	Path	Child-to-father violence							Child-to-mother violence						
		B	SE	95% CI^1^		β	*t*/*z*	*p*	B	SE	95% CI^1^		β	*t*/*z*	*p*
				Lower	Upper						Lower	Upper			
Component	DP ⇒ DU	1.056	0.081	0.898	1.214	0.372	13.103	<0.001	1.048	0.077	0.897	1.199	0.376	13.600	<0.001
	DU ⇒ CPV	0.403	0.052	0.302	0.505	0.243	7.806	<0.001	0.387	0.052	0.284	0.489	0.226	7.401	<0.001
Indirect	DP ⇒ DU ⇒ CPV	0.426	0.108	0.242	0.665	0.090	6.661	<0.001	0.405	0.098	0.240	0.626	0.085	6.516	<0.001
Direct	DP ⇒ CPV	0.702	0.147	0.415	0.990	0.149	4.791	<0.001	0.704	0.146	0.419	0.990	0.148	4.840	<0.001
Total	DP ⇒ CPV	1.128	0.140	0.854	1.402	0.240	8.067	<0.001	1.109	0.138	0.839	1.380	0.233	8.037	<0.001

DP, deviant peer affiliation (predictor); DU, drug use (mediator); CPV, child-to-parent violence (dependent); ^1^ Indirect effect were tested using bias-corrected bootstrap confidence intervals with 10,000 resamples. An indirect effect was considered statistically significant when the 95% confidence interval did not include zero. Z = Statistic of the Sobel Test for the indirect effects and corresponding *p*-value.

Similarly, the child-to-mother violence model (see Table 2) indicates that deviant peer affiliation is directly related to CPV (*β* = 0.148, *p* < 0.001, 95% CI [0.419, 0.990]). A significant indirect effect through drug use was also found (*β* = 0.085, *p* < 0.001, 95% CI [0.240, 0.626]), which explained 36.5% of the total effect of deviant peer affiliation on CPV toward the mother (*β* = 0.233, *p* < 0.001, 95% CI [0.839, 1.380]). Specifically, deviant peer affiliation significantly predicted higher levels of drug use (*β* = 0.376, *p* < 0.001, 95% CI [0.897, 1.199]), which in turn predicted higher levels of CPV toward the mother (*β* = 0.226, *p* < 0.001, 95% CI [0.284, 0.489]).

**Figure 2:**
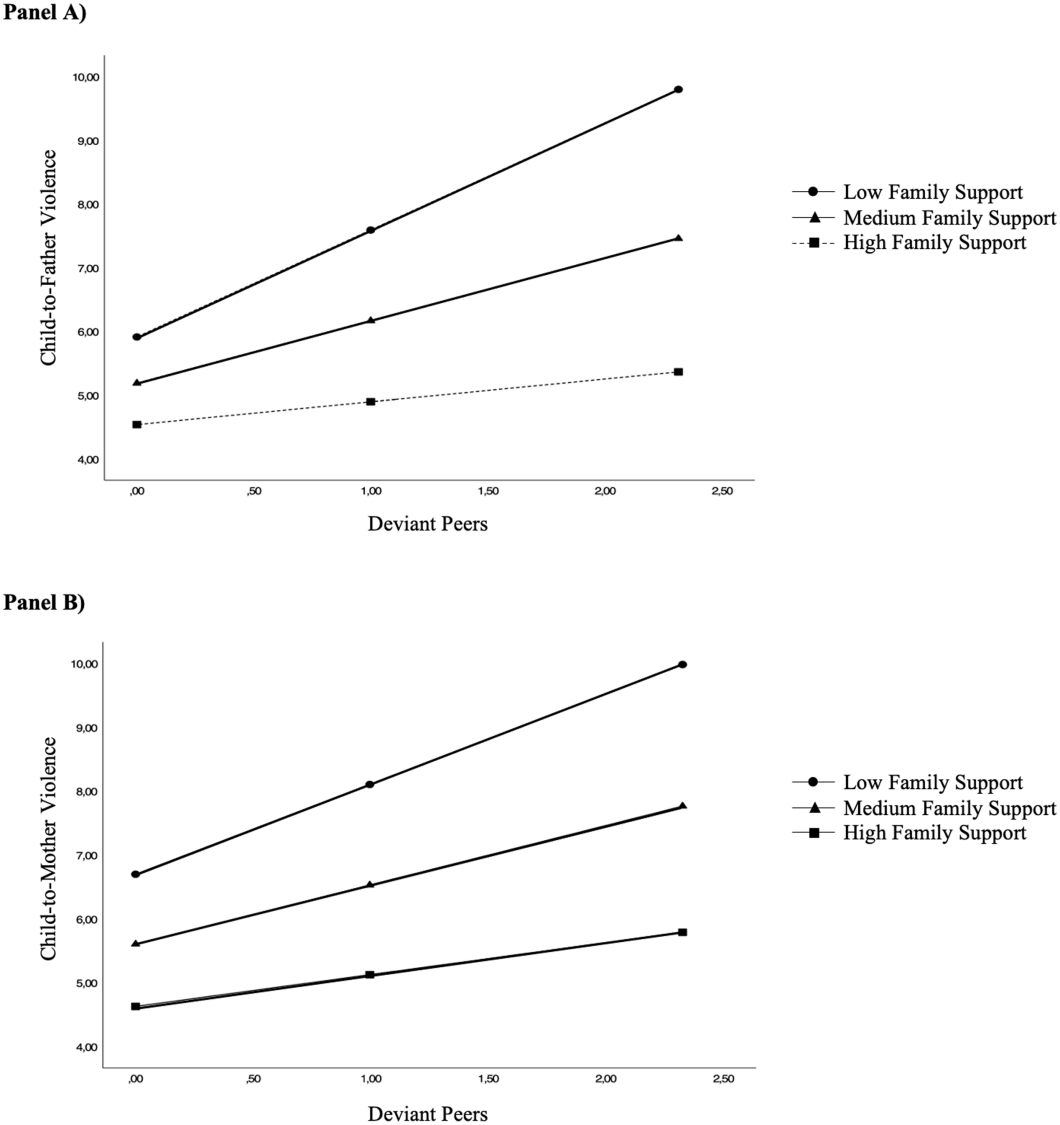
Statistical moderation model. The model for violence towards fathers is presented in panel **(A)**, and the model for the violence towards mothers is presented in panel **(B)**.

Second, the proposed moderation models were tested to examine whether family support moderated the relationship between deviant peer affiliation and CPV. Figure 2 displays the interaction effects (father Panel A and mother Panel B) showing that family support significantly moderates this relationship, reducing its magnitude. Specifically, the results (see Table 3) show, on the one hand, significant main effects of the deviant peer affiliation and family support on CPV toward both parents and, on the other hand, also significant interaction terms between the deviant peer affiliation and family support.

**Table 3:** Coefficients for the moderation models of the relationship between deviant peer affiliation and child-to-parent violence.

Effect	Path	Child-to-Father Violence						Child-to-Mother Violence					
		B	SE	95% CI		*t*	*p*	B	SE	95% CI		*t*	*p*
				Lower	Upper					Lower	Upper		
Main	DP	3.817	0.546	2.746	4.889	6.989	<0.001	2.915	0.541	1.854	3.976	5.391	<0.001
	FS	−0.150	0.046	−0.241	−0.059	−3.244	0.001	−0.228	0.047	−0.320	−0.136	−4.876	<0.001
Interaction	DP * FS	−0.144	0.027	−0.198	−0.090	−5.262	<0.001	−0.101	0.027	−0.154	−0.047	−3.687	<0.001
Conditional	DP at low FS (−1 SD)	1.679	0.182	1.321	2.037	9.209	<0.001	1.411	0.177	1.064	1.758	7.978	<0.001
	DP at mean FS	0.981	0.135	0.716	1.246	7.268	<0.001	0.929	0.134	0.665	1.192	6.917	<0.001
	DP at high FS (+1 SD)	0.358	0.186	−0.007	0.722	1.924	0.055	0.499	0.187	0.131	0.866	2.665	0.008

DP, eviant peer affiliation (predictor); FS, Family support (moderator); SD, Standard deviation.

Analysis of the conditional effects of the child-to-father violence model (see Table 3) indicates that the relationship between deviant peer affiliation and CPV varies according to the level of family support: the association is significant at low levels of family support (*B* = 1.68, *p* < 0.001, 95% CI [0.72, 1.25]), and medium levels (*B* = 0.98, *p* < 0.001, 95% CI [0.72, 1.25]), but not significant at high levels (*B* = 0.36, *p* = 0.055, 95% CI [−0.01, 0.72]). These findings suggest that family support progressively attenuates the negative influence of deviant peer affiliation on CPV toward the father, until it is neutralized at high levels of family support.

In parallel, conditional effects of the child-to-mother violence model (see Table 3) show that the relationship deviant peer affiliation and CPV remains significant at all levels of family support, although its intensity decreases as support increases. Specifically, the effect is strongest at low levels of family support (*B* = 1.41, *p* < 0.001, 95% CI [1.06, 1.76]), moderate with medium levels (*B* = 0.93, *p* < 0.001, 95% CI [0.67, 1.19]), and weaker with high levels (*B* = 0.50, *p* = 0.008, 95% CI [0.13, 0.87]). These results indicate that, although family support progressively reduces the negative influence of deviant peer affiliation on CPV toward the mother, it does not completely neutralize it.

### 3.1 Robustness tests

The robustness tests for mediation (see Table 4) are consistent with the main analyses. In particular, the negative binomial models confirm the direction and significance of the paths *a* (X → M) and *b* (M → Y) from the CPV toward the fathers’ and from the CPV toward the mothers’ models, while the Sobel test confirms the significant indirect effects.

**Table 4:** Robustness test for simple mediation analysis using negative binomial model.

Path	Drug use				Child-to-father violence				Child-to-mother violence			
	*B*	*EE*	IC 95% Wald	***χ***^2^ Wald	*B*	*EE*	IC 95% Wald	***χ***^2^ Wald	*B*	*EE*	IC 95% Wald	***χ***^2^ Wald
DP ⇒ DU	0.194	0.016	0.163, 0.225	149.270^***^								
DP ⇒ CPV					0.109	0.024	0.063, 0.156	21.305^***^	0.093	0.022	0.050, 0.136	18.161^***^
DU ⇒ CPV					0.055	0.008	0.038, 0.072	41.892^***^	0.051	0.008	0.035, 0.066	40.793^***^

Goodness of fit: The Deviance/GL ratio for the models ranged from 1.11 to 1.14, indicating a satisfactory fit; DP, deviant peer affiliation (predictor); DU, drug use (mediator); CPV, child-to-parent violence (dependent); Sobel test for indirect effects: Child-to-Father Violence (*Z* = 5.72, *p* < 0.001); Child-to-Mother Violence (*Z* = 5.70, *p* < 0.001).

***p < 0.001.

Regarding the robustness tests for moderation (see Table 5), the negative binomial model (of both CPV toward the father and CPV toward the mother) also shows main effects consistent with the main analysis in terms of direction as well as of significance. Regarding the interaction term, while the expected direction holds, it does not reach statistical significance. To explore the nature of the interaction, a simple slopes analysis was conducted by segmenting the sample based on the median of the moderator variable (family support). Consistent with the main analysis, the results show that the effect of the deviant peer affiliation on CPV is greater in the low family support group (CPV-Father: *B* = 0.181, *p* < 0.001; CPV-Mother: *B* = 0.148, *p* < 0.001) than in the high family support group (CPV-Father: *B* = 0.120, *p* = 0.002; CPV-Mother: *B* = 0.109, *p* < 0.001). Therefore, both the main analysis and the robustness analysis converge on the direction and magnitude of the interaction effect, although they show differences in the sensitivity of detecting it. This difference could be explained by the fact that, in the main analysis, the nonparametric bootstrap procedure—being more sensitive—allows for the detection of a persistent interaction in the resampling of the data distribution. In contrast, the parametric and more conservative nature of the negative binomial models may limit the model’s statistical power, preventing the interaction from reaching significance under more restrictive distributional assumptions compared to nonparametric procedures.

**Table 5:** Robustness test for simple moderation analysis using negative binomial model.

Path	Child-to-Father Violence					Child-to-Mother Violence				
Global model	*B*	*EE*	IC 95% Wald	***χ***^2^ Wald	*p*	*B*	*EE*	IC 95% Wald	***χ***^2^ Wald	*p*
DP ⇒ CPV	0.142	0.023	0.098, 0.187	39.161	<0.001	0.122	0.021	0.081, 0.163	34.300	<0.001
FS ⇒ CPV	−0.042	0.006	−0.054, −0.030	47.009	<0.001	−0.046	0.006	−0.057, −0.035	65.375	<0.001
DP*FS ⇒ CPV	−0.006	0.004	−0.015, 0.002	1.978	0.160	−0.004	0.004	−0.012, 0.004	0.966	0.326
Simple slopes (Segmentation)										
DP ⇒ CPV (FS_LOW_)	0.181	0.027	0.126, 0.235	42.647	<0.001	0.148	0.024	0.100, 0.196	36.620	<0.001
DP ⇒ CPV (FS_HIGH_)	0.120	0.038	0.044, 0.195	9.691	0.002	0.109	0.036	0.037, 0.181	8.753	0.003

Goodness of fit: The Deviance/GL ratio for the models ranged from 1.13 to 1.14, indicating a satisfactory fit; DP, Deviant peer affiliation (predictor); FS, Family support (moderator); CPV, child-to-parent violence (dependent).

## 4 Discussion

This study was aimed to analyze, in a sample of non-emancipated young adults, whether drug use mediates the relationship between the deviant peer affiliation and the violence towards fathers and mothers, as well as to examine whether family support moderates this relationship.

In hypothesis 1 it was expected that deviant peer affiliation would be positively and directed related to CPV, and indirectly more strongly through drug use. The results confirmed this hypothesis. First, the data revealed that deviant peer affiliation is significantly and directly related to CPV, which is consistent with previous literature on this topic (Contreras and Cano-Lozano, 2015; Loinaz and de Ma Sousa, 2020). Furthermore, results indicated that deviant peer affiliation is related to drug use. This relationship has also been previously demonstrated (e.g., Duan et al., 2009; Fergusson et al., 2002; Kendler et al., 2014), together with the association between drug use and CPV (e.g., Armstrong et al., 2018; Beckmann et al., 2017). Notwithstanding, the current study go further by showing, in a sample of young adults, a significant indirect effect between deviant peer affiliation and CPV through drug use. That is, deviant peer affiliation was positively related to drug use, which in turn was positively related to CPV toward the father and the mother. These results are in line with those by Cano-Lozano et al. (2020) and Del Hoyo-Bilbao et al. (2020) with samples of adolescents. The affiliation with deviant peers (friends who use drugs, exhibit violent behavior, or have been involved in criminal activity) increase the likelihood of drug use. Following to Hinnant et al. (2022), peers exert a powerful influence on behavior and associations with deviant peers may increase access to illicit substances and contribute to the reinforcement of substance use behaviors. In addition, drug use is well known as a source of conflict with parents (Svensson et al., 2019). This could be either because its effects promote disinhibited behaviors that, as indicated by Pagani et al. (2009), in confrontations with parents would increase the likelihood of aggression toward them or because the use of violence with instrumental reasons (as for example to get money from parents to get drugs). However, given the complexity of these relationships, it is not clear if the association with deviant peers promote CPV behaviors in young adults who use drugs or if it simply supports the antisocial lifestyle that they exhibit. This issue raises the question about if these violent young people tend to associate with those similar or if deviant peers influence their violent behaviors towards parents. Following to Ragan (2019) two processes explain this relationship. The first mechanism, referred to as “peer influence” or “peer socialization,” refers to the process by which individuals progressively adopt the beliefs, attitudes, or behaviors of those with whom they associate. The second mechanism, named “selection process” describes the tendency for social ties, particularly friendships, to form between individuals who share similar characteristics. In this context, individuals engaged in antisocial behavior preferentially affiliate with others who exhibit comparable behavioral profile. In this line, in classical interactional theory of delinquency (Thornberry, 1987; Thornberry et al., 1994) the association between delinquent peers and delinquent behavior is conceptualized as reciprocal and dynamic, and recent evidence continues to support this bidirectional relationship (Cho, 2021; Kim and Lee, 2023). Affiliation with delinquent peers increases the likelihood of engaging in delinquent behavior, which subsequently fosters further associations with similarly delinquent peers, thereby reinforcing and perpetuating delinquency over time.

In hypothesis 2, family support was expected to buffer the positive relationship between deviant peer affiliation and CPV. Our results confirmed this hypothesis, as family support significantly moderated this relationship, reducing its magnitude. Concretely, the relationship between deviant peer affiliation and CPV varies according to the level of family support, as it progressively attenuates the influence of deviant peer affiliation on CPV and, in the case of child-to-father violence, it is even neutralized at high levels of family support. Overall, the findings indicate that high levels of family support function as a protective factor against CPV, attenuating the impact of affiliation with deviant peers. Specifically, high levels of family support appear to neutralize the negative influence of deviant peers on violence directed toward the father and to significantly reduce their effect on the development of violence toward the mother. This implies that, in the presence of deviant peers, family support may be a barrier to their negative influence. This is also consistent with previous literature that reveals that family support acts as a moderator between different risk factors and several problems during adolescence (Gao et al., 2020; Kennedy et al., 2010; Ozer, 2005; Schofield et al., 2012; Trudeau et al., 2012). In the same line, the study by Gao et al. (2013) with adolescents found that a healthy family functioning moderated the relationship between deviant peers and delinquency, decreasing the influence of deviant peer association. These results suggest that, although the influence of the family during adolescence and early adulthood may diminish (in favor of the influence of the peer’s group), the family environment continues to exert a significant protective effect by reducing youths’ likelihood of engaging in antisocial and delinquent behaviors.

This study has several limitations that should be considered when interpreting the findings. First, the data rely exclusively on young adults’ self-reports, which may have introduced social desirability biases, particularly given the sensitive nature of CPV and deviant peer affiliation. Future studies would benefit from incorporating parents’ reports to obtain a more comprehensive understanding of family dynamics and peer influences. Second, the use of snowball sampling and online data collection may have introduce selection bias, as participants were recruited through personal networks. This procedure could have led to the overrepresentation of certain demographic characteristics, thereby limiting the representativeness and generalizability of the results. Third, the cross-sectional design precludes conclusions about developmental direction of the associations observed. This limitation is especially relevant considering that the relationship between deviant peer affiliation and CPV has been conceptualized as dynamic and reciprocal in previous theorical models. Therefore, longitudinal studies are needed to clarify temporal ordering and stability of these relationships during emerging adulthood. On the other hand, although the main analytical approach is appropriate for estimating robust interaction effects, the negative binomial model does not fully confirm a statistically significant interaction term, suggesting that this effect should be interpreted with caution pending further replication. Finally, although the sample size was large, participants were drawn from different regions of Spain and shared a specific cultural and social context. This issue may be particularly relevant in studies of CPV among emerging adults, as family relationships and transitions to adulthood differ across cultures. In Southern European countries such as Spain, prolonged co-habitation with parents, strong parental involvement during emerging adulthood and strong family interdependence are relatively common (García-Mendoza et al., 2022, 2024). These cultural aspects may increase opportunities for parent–child conflicts and shape the influence of deviant peers differently than in more individualistic contexts characterized by earlier residential independence. Therefore, caution is warranted when generalizing the results to other populations with different values or family structures.

Despite these limitations, this is the first study that examine the influence of deviant peer affiliation on CPV in a sample of non-emancipated young adults, as the scarce research on this issue has been conducted with adolescents. Thus, much less is known about whether these associations operate similarly during emerging adulthood. Examining these relationships in emerging adults is particularly relevant because developmental changes associated with adulthood such as greater autonomy, changes in family relationships or more stable peers’ affiliation (Marzilli et al., 2021; Samek et al., 2016) may modify the dynamics underlying CPV. Moreover, the persistence of CPV beyond adolescence could reflect a more severe and chronic pattern of violence (Kessler, 2020), underscoring the need to examine whether theorical frameworks and previous studies with adolescent population remain applicable in adult samples. Furthermore, our results provide additional evidence about the complexity of the pathways between the affiliation with deviant peers and CPV, examining the mediating role of drug use and the moderating role of family support in this relationship. Future studies could expand on the literature by addressing other protective factors that may interrupt these paths, incorporating, for example, some cognitive and emotional variables (such as empathy, personal values, copying styles, etc.) which might help to explain the complexity of these relationships. Recent studies reveal that CPV is also related to other forms of youth violence, such as dating violence and bullying (Contreras et al., 2026; Navas-Martínez et al., 2025b; Zhang et al., 2025) so prevention strategies are crucial for limiting the emergence of future patterns of violent behavior. Consequently, in terms of practical implications, these findings highlight the relevance of further examining social variables that have traditionally received limited attention in CPV research, such as affiliation with deviant peers. They also underscore the importance of early preventive interventions, particularly within educational settings, which may represent an appropriate context for identifying and addressing these risk factors. It is also remarkable the need of continuing to design or improve specific prevention programs targeting substance use, as well as antisocial and violent behaviors, by incorporating recent evidence on factors involved in the onset and maintenance of CPV. In light of the present findings, the existing prevention programs could be enhanced by incorporating strategies designed to improve family support and family cohesion, concretely by including family-focused components aimed to strengthening parent-youth communication, emotional support and positive family relationships. Promoting family support may reduce the young people’ vulnerability to deviant peer influence and related antisocial behaviors, thereby acting as a protective factor against CPV.

## Data Availability

The raw data supporting the conclusions of this article will be made available by the authors, without undue reservation.
